# Notes and News

**Published:** 1904-08-27

**Authors:** 


### Notes and News.

Beri-beri accounted for more cases of sickness in the United States army in 1903 than typhoid.

A new operating theatre has been opened at the Chester General Infirmary, which has been presented by Mrs. R. Tidswell at a cost of £600. The gift is in memoriam of the late Mr. R. Tidswell.

Professor Koch states that experiments have been carried out by the German Imperial Institute of Health which corroborate his theory that human and bovine tuberculosis are different from one another, and that the work will be published in detail in a few months.

A fever hospital for Lower Banffshire has been opened at Portsoy. Dr. Campbell, of Old Cullen, contributed no less than £4,000 to defray the cost of the building, and Lady Seafield granted a site of two acres practically free of cost. The hospital, which is named the Campbell Hospital, contains 20 beds.

Chicago has made an advance in the treatment of the insane by the establishment of cottage residences for the reception of patients. They are arranged on the lines of ordinary private houses and are constructed in a picturesque style, all arrangements being towards cheerfulness and brightness, with an absence of any appearance of imprisonment.

Readers of the *New York Medical Record* will receive the announcement of the retirement of the editor, Dr. George Shrady, with regret. For 38 years Dr. Shrady has conducted the paper with the greatest efficiency and zeal. His ideals have been high ones, and we feel that he must look back upon his work with satisfaction. His journalistic occupations did not absorb all his interest. He did much good professional work, and many sanitary improvements in New York owe themselves to him. His kindness will not be forgotten by many struggling young journalists and others whom he came across battling with the difficulties of earning a livelihood. He retires from the post he has adorned nearly the veteran amongst medical editors throughout the world. As far as we know, the *Lancet* alone has possessed an editor of longer standing.

Sir William Bennett, K.C.V.O., F.R.C.S., has received the Gold Cross of the Royal Order of the Saviour from the King of Greece.

It is reported that the Viceroy of the Province of Sze Chuen is arranging to open a Chinese medical school, with French instructors. Apparently the school is to have an especial military object, as members of the Chinese army are to be selected and sent for instruction.

The foundation-stone has been laid of an isolation hospital for fever patients at Rotherham. It will provide 18 beds for scarlet fever, 16 beds for mild cases of the same disease, 14 beds for typhoid fever, 14 for diphtheria, and 14 for cases under observation. There will be a veranda in front of each ward.

The matron of the Leper Asylum near Pretoria, who is about to return to her post, is appealing for articles to brighten the lot of the patients. A magic lantern, books, games, pictures, toys, etc., she states, would be welcomed. Messrs. Bullard, King, and Co., East India Docks, will take charge of packets addressed to Miss Whiteman, and marked for the "Lepers at Pretoria." The asylum ministers to about 300.

Philadelphia is taking most drastic steps to prevent the introduction or spread of small-pox. Fifty inspectors are employed on house-to-house visitations to canvass for vaccination. Those who refuse to be vaccinated are registered, and employers and physicians notified, the employer being further warned that one case of small-pox occurring on the premises will cause an establishment to be closed for fumigation.

Sir Felix Semon, who will deliver the address on Laryngology at the St. Louis Congress of Arts and Sciences in September, will be the guest of Professor G. Lefferts and Dr. Emil Mayer, who are both associated with him on the *Internationale Centralblatt für Laryngologie*, of which he has been editor-in-chief for 20 years. Sir Felix Semon appears likely to make a most interesting and pleasant tour in America and Canada, considerable preparation having been made to entertain him.

The David Lewis Manchester Colony for Epileptics is nearly completed. The money offered originally and declined by the Manchester St. Mary's and Southern Hospitals to form a combined institution has been devoted to the establishment of this colony; and considering how great is the need of proper care for epileptics, their decision can hardly be regretted in the interests of the community in general. The sum expended has amounted to about £120,000. The situation, near Alderley Edge and Chelford, in Cheshire, is very suitable. About 130 acres are available, and on this site are erected the numerous buildings to house the colony. There are three homes for men, three for women, and a school for children, and several cottages also. Altogether 200 patients can be received, and these will receive the care and occupation suitable to their condition. Payments will be required; but these cannot meet the expenses to any great degree, and so subscriptions and donations will be much needed.

